# Localized cytokine responses to total knee arthroplasty and total knee revision complications

**DOI:** 10.1186/s12967-020-02510-w

**Published:** 2020-08-31

**Authors:** Nicole Prince, Julia A. Penatzer, Matthew J. Dietz, Jonathan W. Boyd

**Affiliations:** 1 C. Eugene Bennett Department of Chemistry, West Virginia University, 64 Medical Center Drive, 3900 HSS, P.O. Box 9196, Morgantown, WV 26506-9196 USA; 2 Department of Orthopaedics, West Virginia University School of Medicine, Morgantown, WV USA; 3 Department of Physiology and Pharmacology, West Virginia University, Morgantown, WV USA

**Keywords:** Interleukin, Infection, Inflammation, Total knee arthroplasty, Total knee revision, Spatial distribution

## Abstract

**Background:**

The study of localized immune-related factors has proven beneficial for a variety of conditions, and one area of interest in the field of orthopaedics is the impact of implants and localized infections on immune response. Several cytokines have shown increased systemic concentrations (in serum/plasma) in response to implants and infection, but tissue-level cytokines have not been investigated as thoroughly.

**Methods:**

This exploratory study investigated tissue-level cytokines in a cohort of patients (N = 17) in response to total knee arthroplasty and total knee revision to better understand the immune response to implants and localized infection (e.g., prosthetic joint infection). The overall goal of this study was to provide insight into the localized cytokine response of tissues and identify tissue-level markers specific to inflammation caused by implants vs. inflammation caused by infection. Tissues were collected across several anatomical locations and assayed with a panel of 20 human inflammatory cytokines to understand spatial differences in cytokine levels.

**Results:**

In this study, six cytokines were elevated in implanted joints, as compared to native joints: IL-10, IL-12p70, IL-13, IL-17A, IL-4, and TNF-α (p < 0.05). Seven cytokines showed infection-dependent increases in localized tissues: IL-1α, IL-1β, IL-6, IL-8, MCP-1, MIP-1α, and MIP-1β (p < 0.05).

**Conclusions:**

This study demonstrated that differences exist in tissue-level cytokines in response to presence of implant, and some cytokines were specifically elevated for infection; these responses may be informative of overall tissue health. These results highlight the utility of investigating localized cytokine concentrations to offer novel insights for total knee arthroplasty and total knee revision procedures, as well as their complications. Ultimately, this information could provide additional, quantitative measurements of tissue to aid clinical decision making and patient treatment options.

## Background

The inflammatory response of tissues involves a series of biological events regulated by a number of immune factors, and the actions of these immune factors are partially reliant on the cytokines and chemokines produced in response to pathogens, foreign bodies, and other stimuli [1–3]. These responses are of interest to the field of orthopaedics, especially with regard to the immune response to implants, infection, and chronic inflammation [4–6]. An elevated immune response has been observed following total knee arthroplasty (TKA) procedures; increased levels of cytokines, particularly interleukin (IL)-1, IL-4, IL-6, IL-10, and tumor necrosis factor alpha (TNF-α), have been shown both on a systemic level (i.e., serum/plasma) as well as on a more localized level (i.e., synovial fluid) [7–9]. However, many aspects of this response are not well understood. For example, a majority of TKA procedures are successful, but implant-related and infection-related complications can negatively affect a patient’s quality of life. Properly addressing these issues is of high priority to the field of orthopaedics, especially considering the increasing demand for joint replacement [10]. Many studies have noted the pain, inflammation, and dissatisfaction that can occur following these procedures, affecting approximately 20% of patients undergoing TKA [11, 12], but it is not entirely known what role cytokines play in this chronic inflammatory response. Infections, such as prosthetic joint infection (PJI), are another serious complication and are a leading cause of total joint failure [13]. PJI is a localized infection surrounding a prosthetic joint and can result following implantation, often necessitating surgical intervention [14]. PJI is a major concern following TKA/total knee revision (TKR) procedures and can be difficult to treat. The infections are often persistent and unable to be resolved using conventional methods, presenting a challenge for clinicians [15]. The systemic immune response to PJI has been studied extensively, but the localized tissue response is not as well understood. In order to better understand the immune response to implants and localized infection, this study investigated levels of 20 inflammatory cytokines in localized tissue surrounding the joint. While defining the localized response to implants and infection can be difficult [7–9], localized cytokine responses have been investigated for other pathological conditions. A few studies have characterized localized cytokine responses in trauma [16–18] and respiratory infection [19], and these studies demonstrated that the local cytokine environment differs when compared to systemically circulating levels. Currie et al. [16] showed that differences in cytokine concentrations exist in skeletal muscle samples in a spatially-dependent manner using an animal model of traumatic injury. Similarly, Hauser et al. [18] observed differences in levels of cytokines at the site of injury compared to systemic levels in response to trauma in humans. Other research groups have observed spatially-related differences of other immune-related factors for stroke [20], and in response to allergens [21] in animal models. These studies introduced the concept of using immune markers on a localized level to better understand these conditions.

TKA and TKR procedures trigger inflammatory cascades, initiating cytokine responses and elevating systemic cytokine concentrations; higher levels of cytokines have been observed following these surgeries. The elevation in cytokine levels has been attributed to the trauma of surgery as well as the introduction of implants into the body [22, 23]. However, this inflammation is sometimes prolonged, which can cause major complications for patients. The causes of chronic inflammation following these procedures are still unknown, and resolution of the inflammation is challenging [24]. Therefore, understanding the changes in inflammatory response specific to implant-related inflammation is beneficial to improving the outcome of these individuals.

Similarly, the localized response to PJI has not been characterized to understand the local immune modulation in these cases. Many studies have investigated systemically circulating levels of interleukins and other cytokines for their roles in infection, and several cytokines are used as diagnostics of PJI [25–27]. Several studies have specifically focused on the utility of measuring IL-6 and IL-8 levels in serum for diagnosing and monitoring PJI, both of which have increased specificity over conventional methods; this knowledge has greatly benefitted the clinical treatment options for PJI [28, 29]. However, PJI remains one of the most serious complications following revision knee arthroplasty. In fact, infection is one of the most common causes for revision, being implicated in 20.4% of all revision TKA procedures between 2009 and 2013 [30]. While defining the systemic response to sepsis and infection has paved the way for improved diagnostics [31–33], less is known about the environment of localized infections and what role cytokines play in determining tissue health.

The present study focused on understanding differences in localized distributions of cytokines in TKA and TKR procedures, with and without presence of infection, using PJI as the model for localized infections. The ultimate goal of this study was to characterize the immune modulation on a tissue level that occurs in response to joint implantation and infection to better understand localized tissue health. The information gained could aid clinical management of these complications by narrowing down cytokines that are indicative of response to PJI. It represents the first known investigation of tissue-level cytokines in response to implant-related and infection-related complications, to our knowledge.

## Patients, materials, and methods

### Patients

Following Institutional Review Board (IRB) approval (IRB Protocol #1709745853) and patient consent, six patients undergoing primary total knee arthroplasty (TKA) and 11 patients undergoing total knee revision (TKR) procedures participated in the study (8 males, 9 females; aged 45–82 years; body max index [BMI] 24.6–43.7). Subjects were recruited over a 12-month period. All six primary TKA patients were undergoing elective surgery for total replacement of the knee joint with a diagnosis of osteoarthritis. At the time of this study, this was the first arthroplasty procedure on either knee joint. In the TKR group, patients were further characterized into aseptic and septic revision procedures. Patients with aseptic revisions (N = 5) were undergoing revisions due to failures of the prosthetic joint but did not show presence of infection. For ease of the reader, samples from these patients will be referred to as aseptic TKR tissues. Patients with septic revisions (N = 6) met clinical criteria for a PJI diagnosis as defined by the Musculoskeletal Infection Society (MSIS) criteria [13]. Samples from these patients will be referred to as septic TKR tissues. All six patients diagnosed with PJI were tissue culture positive: four tested culture positive for *Staphylococcus epidermidis*, one for Methicillin-sensitive *Staphylococcus aureus* (MSSA), and one for *Enterobacter cloacae*. More patient information can be found in Table 1. Systemic C-reactive protein (CRP) levels in serum are additionally listed as reference.

**Table 1:** Patient information

ID	Sex	TKA/TKR	BMI (kg/m^2^)	Diabetic (Y/N)	CRP (mg/L)	Culture
P1	F	TKA	33.8	N	N/A	Negative
P2	F	TKA	39.8	N	N/A	Negative
P3	F	TKA	39.8	N	N/A	Negative
P4	M	TKA	29.7	Y	N/A	Negative
P5	M	TKA	24.6	N	N/A	Negative
P6	M	TKA	27.2	N	N/A	Negative
F1	F	TKR-aseptic	28.2	N	4.3	Negative
F2	F	TKR-aseptic	29.8	N	0.2	Negative
F3	F	TKR-aseptic	33.9	N	< 1	Negative
F4	M	TKR-aseptic	40.4	Y	3.6	Negative
F5	M	TKR-aseptic	26.2	N	2.1	Negative
F6	F	TKR-septic	43.7	N	28.8	*S. epidermidis*
F7	F	TKR-septic	30.8	Y	161.4	*S. epidermidis*
F8	F	TKR-septic	41.9	N	21.7	*E. cloacae*
F9	M	TKR-septic	36.2	N	33.5	*MSSA*
F10	M	TKR-septic	33.8	Y	3.8	*S. epidermidis*
F11	M	TKR-septic	31.9	N	111.9	*S. epidermidis*

Six primary TKA and 11 revision TKR patients were enrolled in the study, creating a heterogenous cohort of males and females varying in age (45–82 years) and comorbidities. Primary TKA patients have ID format P#; revision TKR patients have ID format F#. This table lists general patient information including the pathogen for which each septic patient tested culture-positive following testing on the day of surgery. Serum CRP values were obtained pre-operatively in the revision setting. Cultures were obtained from intraoperative tissue samples

### Collection of tissue samples

**Fig. 1:**
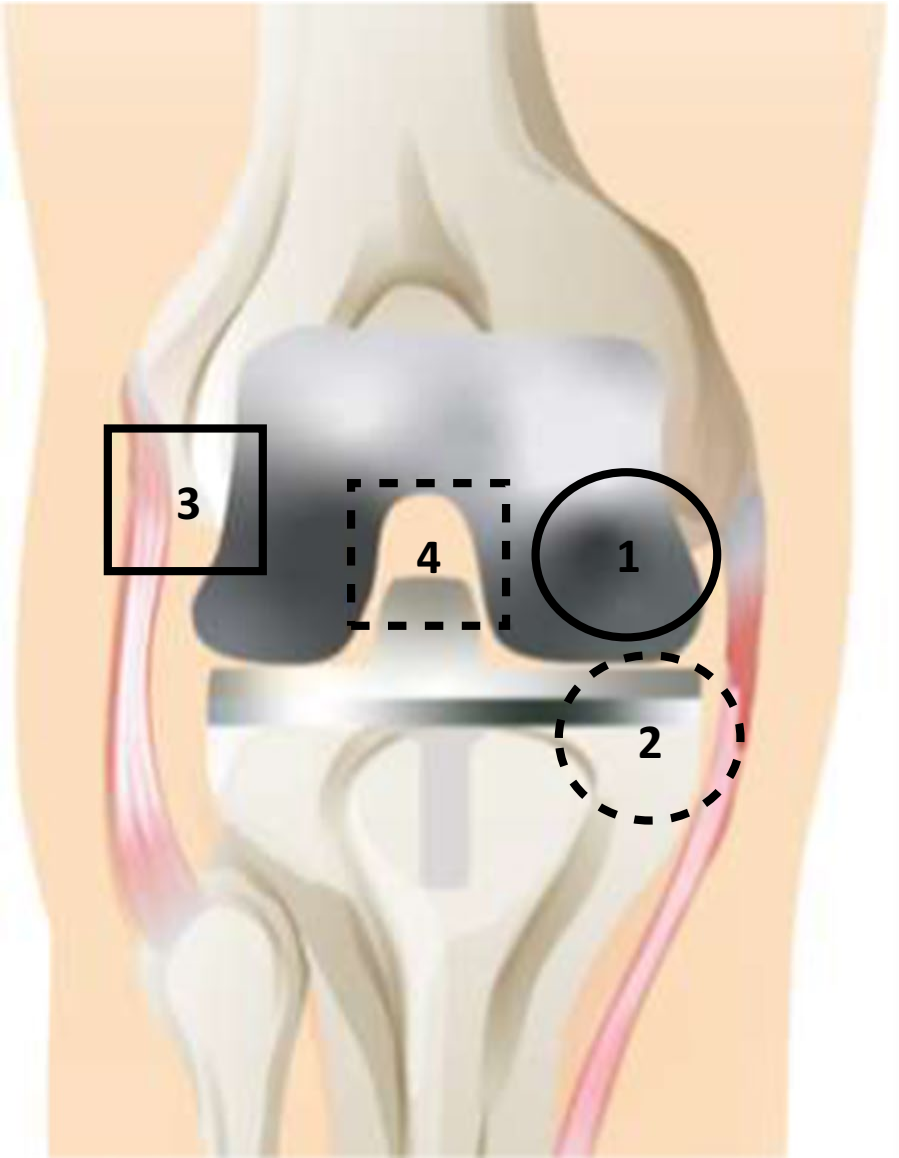
Map of approximate tissue collection locations, shown with prosthetic implant illustrated. Seven tissue samples were taken for each patient; (1) the solid circle represents the medial femoral condyle (denoted as F); (2) the dashed circle represents the medial tibial plateau (denoted as T); (3) the solid square represents the lateral gutter (denoted as LG); (4) the dashed square represents the posterior capsule (denoted as PC). Locations 1–4 were taken for the ATL layer, and locations 1–3 were taken for the RTL layer; separation between ATL (closer to joint) and RTL (further from joint) was approximately 5–10 mm, depending on individual patient

All TKA and TKR procedures were performed by a single surgeon with standard debridement and washing protocols. Tissues were collected at a total of four distinct anatomical locations, broadly characterized into two tissue layers: four adjacent tissue layer (ATL) samples and three radial tissue layer (RTL) samples. The ATL samples came from the initial debridement. Tissues from the ATL layer were closer to the knee joint (or prosthetic implant). Conversely, RTL samples were taken from a tissue layer further removed from the joint (or prosthetic implant) after the surgeon completed debridement. The difference in depth of the RTL tissues and ATL tissues was approximately 5–10 mm and was dependent on the individual patient. Measurements were made from point of origin to standardize tissue samples taken between patients. Tissues were taken at four anatomical locations illustrated in Fig. 1. Briefly, the solid line circle represents location (1) medial femoral condyle (F); the dashed line circle represents location (2) medial tibial plateau (T); the solid line square represents location (3) lateral gutter (LG); and the dashed line square represents location (4) posterior capsule (PC). Anatomical locations 1–4 were collected for the ATL layer, and locations 1–3 were collected for the RTL layer. Location 4, PC, could not be taken in the RTL layer due to proximity to neurovascular structures. Therefore, a total of seven tissue samples were taken for each patient.

### Sample preparation

Tissues were collected during TKA and TKR procedures in the operating room and immediately stored on dry ice. Once all tissues had been collected for an individual patient, they were washed with 1× cold phosphate-buffered saline (PBS) to remove blood and debris. Tissues were grossly dissected using a scalpel to remove scar tissue or cement, then stored at − 80 ºC. When samples had been collected for all patients, tissues were thawed on ice and cut into sections approximately 30 mg in size; tissues were homogenized by sonication in 500 µL cell lysis solution (Bio-Rad, Hercules, CA) containing 20 mM phenylmethylsulfonyl fluoride (Sigma-Aldrich, St. Louis, MO). Protein extraction was performed using methods adapted from Hulse et al. [34]. Thawed samples were vortexed for 1–3 s and centrifuged at 5000×*g* for 5 min at 4 °C. The supernatant was collected and tested for total protein content using a Pierce BCA Protein Assay Kit (Thermo Scientific, Waltham, MA), according to manufacturer’s instructions. Absorbance values for total protein content were determined on an Infinite M1000 multimode plate reader (Tecan, Raleigh, NC).

### Cytokine measurement

To standardize samples for total protein content, tissue homogenates were individually diluted to a total protein concentration of 900 µg/mL with cell lysis buffer (Bio-Rad). Cytokine quantification was performed using a magnetic bead-based multiplex Inflammation Human ProcartaPlex panel assay (Invitrogen, Carlsbad, CA) and measured using a Bio-Plex 200 suspension array system and Pro II Wash Station (Bio-Rad), according to the manufacturer’s instructions.

### Statistical analysis

Data were analyzed using Prism 5 (GraphPad, San Diego, CA) and SAS JMP (Cary, NC). Standard curves were generated for each protein using either a four- (4PL) or five-parameter logistic (5PL) regression model, depending on the individual protein. Cytokine concentrations were determined using standard curve interpolation, then corrected by dilution factor to compare tissue homogenates. Cytokine concentrations are expressed as picograms of cytokine per milliliter of tissue homogenate (pg/mL). Samples with fluorescence intensity values below the lower limit of quantitation (LLOQ) or above the upper limit of quantitation (ULOQ) were omitted from statistical comparisons. Outliers were identified using the 1.5× interquartile range (IQR) rule and omitted from analysis. Two-way analysis of variance (ANOVA) with Bonferroni’s post-test was used to determine significant differences between primary TKA, aseptic TKR, and septic TKR tissue samples at each tissue location. Each tissue homogenate was tested in duplicate for cytokine concentration. Data are expressed as the mean ± standard error of the mean (SEM).

Quadratic discriminant analysis was conducted to evaluate the combined capacity of cytokine response to predict the state of tissue. Using SAS JMP, all measured responses were cast as covariates, and the “group” was assigned as a classification category (primary TKA, aseptic TKR, septic TKR). The *Shrink Covariances* option was applied to account for the different covariances within the categories. Quadratic discriminant analysis is a predictive modeling tool, and when there are a large number of variables compared to observations, as is the case in this study, *Shrink Covariances* is frequently employed to improve the stability and reduce prediction variance [35]. This analysis included 13 covariates; only those cytokines that produced statistically significant two-way ANOVA comparisons for either infection-specific or implant-specific comparisons were included: IL-1α, IL-1β, IL-6, IL-8, monocyte chemoattractant protein (MCP)-1, macrophage inflammatory protein (MIP)-1α, MIP-1β, IL-10, IL-12p70, IL-13, IL-17A, and TNF-α. Biplot rays are plotted to indicate how each covariate influences the canonical space, with the direction and magnitude signifying the degree of association with the respective group (primary TKA, aseptic TKR, septic TKR).

Due to the limited sample size, this study was not able to control for age, sex, BMI, or other comorbidities. Pearson correlations were run between cytokine concentrations and age, sex, and BMI for each patient to analyze the contribution of these variables. Bonferroni’s correction was applied to correct for multiple inferences, as previously described by Bland et al. [36].

## Results

Changes in cytokine concentrations were observed for comparisons of primary TKA vs. aseptic TKR vs. septic TKR tissues. Overall, cytokine concentrations were generally elevated in TKR (both septic and aseptic) compared to TKA, and septic TKR exhibited higher cytokine levels than aseptic TKR for several cytokines. Seven cytokines (IL-1α, IL-1β, IL-6, IL-8, MCP-1, MIP-1α, and MIP-1β) showed increased concentrations in septic TKR tissues compared to both aseptic TKR tissues and primary TKA tissues (p < 0.05). Six cytokines (IL-10, IL-12p70, IL-13, IL-17A, IL-4, and TNF-α) showed differences in concentration between primary TKA and TKR (both aseptic and septic) (p < 0.05), but these six cytokines were not significantly different between aseptic TKR and septic TKR. These comparisons are described in detail over the following sections. Additional human inflammatory cytokines were tested, but they did not produce statistically significant comparisons in this study: E-Selectin, granulocyte–macrophage colony-stimulating factor (GM-CSF), interferon-alpha (IFN-α), interferon-gamma (IFN-γ), and interferon gamma-induced protein 10 (IP-10).

### Seven cytokines exhibited infection-specific elevation in localized tissues

**Fig. 2:**
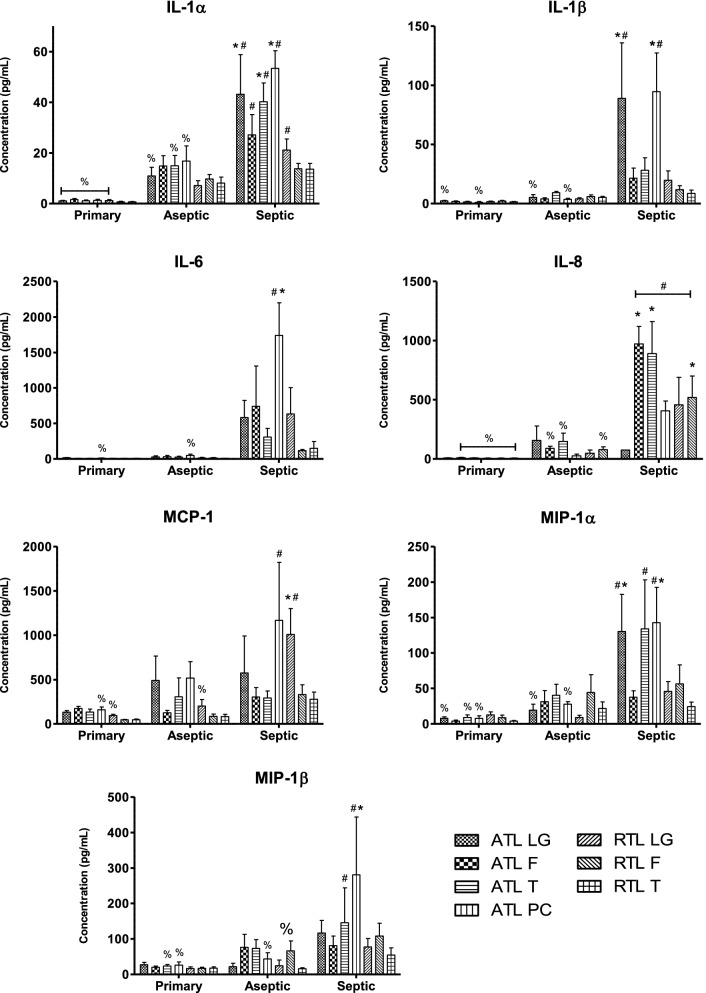
Seven cytokines showed infection-dependent elevation in localized tissues. Individual tissue locations are shown for all groups. Two-way ANOVAs with Bonferroni’s post-test were conducted to test for significant differences between groups at each individual location (p < 0.05). Significant differences between groups at a particular location are marked as: * denotes significant difference from primary TKA (N = 6); # denotes significant difference from aseptic TKR (N = 5); % denotes significant difference from septic TKR (N = 6); all symbols denote significance at the p < 0.05 level

Seven cytokines showed an increase in concentration that was dependent on the presence of localized infection: IL-1α, IL-1β, IL-6, IL-8, MCP-1, MIP-1α, and MIP-1β (p < 0.05). For these cytokines, primary TKA averages were lowest, with an increase in aseptic TKR and further increase in septic TKR. For IL-1α, the average concentration of primary TKA tissues was 1.1 pg/mL, and rose to 11.8 pg/mL in aseptic TKR; the concentration was elevated to 30.3 pg/mL in septic TKR. Location-specific differences are marked in Fig. 2, and it is clear that most significant comparisons are present within locations of the ATL layer. IL-1β showed a similar trend, with a mean of 1.7 pg/mL in primary TKA tissues, which rose to 5.4 pg/mL in aseptic TKR, and further elevated to 39.1 pg/mL in septic TKR. For IL-1β, the most marked differences between groups came from comparisons of locations ATL LG and ATL PC (Fig. 2). IL-6 followed, with an average of 8.5 pg/mL in primary TKA, rising to 24.2 pg/mL in aseptic TKR, and finally 610.7 pg/mL in septic TKR. Location ATL PC showed the most dramatic increase in concentration in septic TKR compared to other groups (Fig. 2). IL-8 levels were 7.6 pg/mL in primary TKA, which increased to 91.1 pg/mL in aseptic TKR, and rose to 553.9 pg/mL in septic TKR. Differences in IL-8 were significant between at least two groups at all locations besides ATL LG and ATL PC at p < 0.05 (note: ATL PC for IL-8 not shown due to omission of outliers). For MCP-1, the average of primary TKA tissues was 113.0 pg/mL, which increased to 258.8 pg/mL for aseptic TKR, and further increased to 565.1 pg/mL for septic TKR. RTL LG showed the most significant comparisons between groups for MCP-1 (Fig. 2, p < 0.05). MIP-1α followed the same trend, with an average of 7.8 pg/mL for primary TKA, which rose to 27.8 pg/mL in aseptic TKR, and was elevated to 81.6 pg/mL in septic TKR. ATL locations showed the most significant increases in MIP-1α between groups (Fig. 2, p < 0.05). For MIP-1β, primary TKA tissues showed an average of 21.3 pg/mL and were increased to 46.0 pg/mL for aseptic TKR and further increased to 123.4 pg/mL in septic TKR. Locations ATL T and ATL PC showed the most significant increases between groups for MIP-1β (Fig. 2, p < 0.05). As shown in Fig. 2, cytokine concentrations in the ATL layer locations were generally higher than the RTL layer locations.

### Six cytokines exhibited implant-related elevation (primary TKA vs. aseptic/septic TKR)

**Fig. 3:**
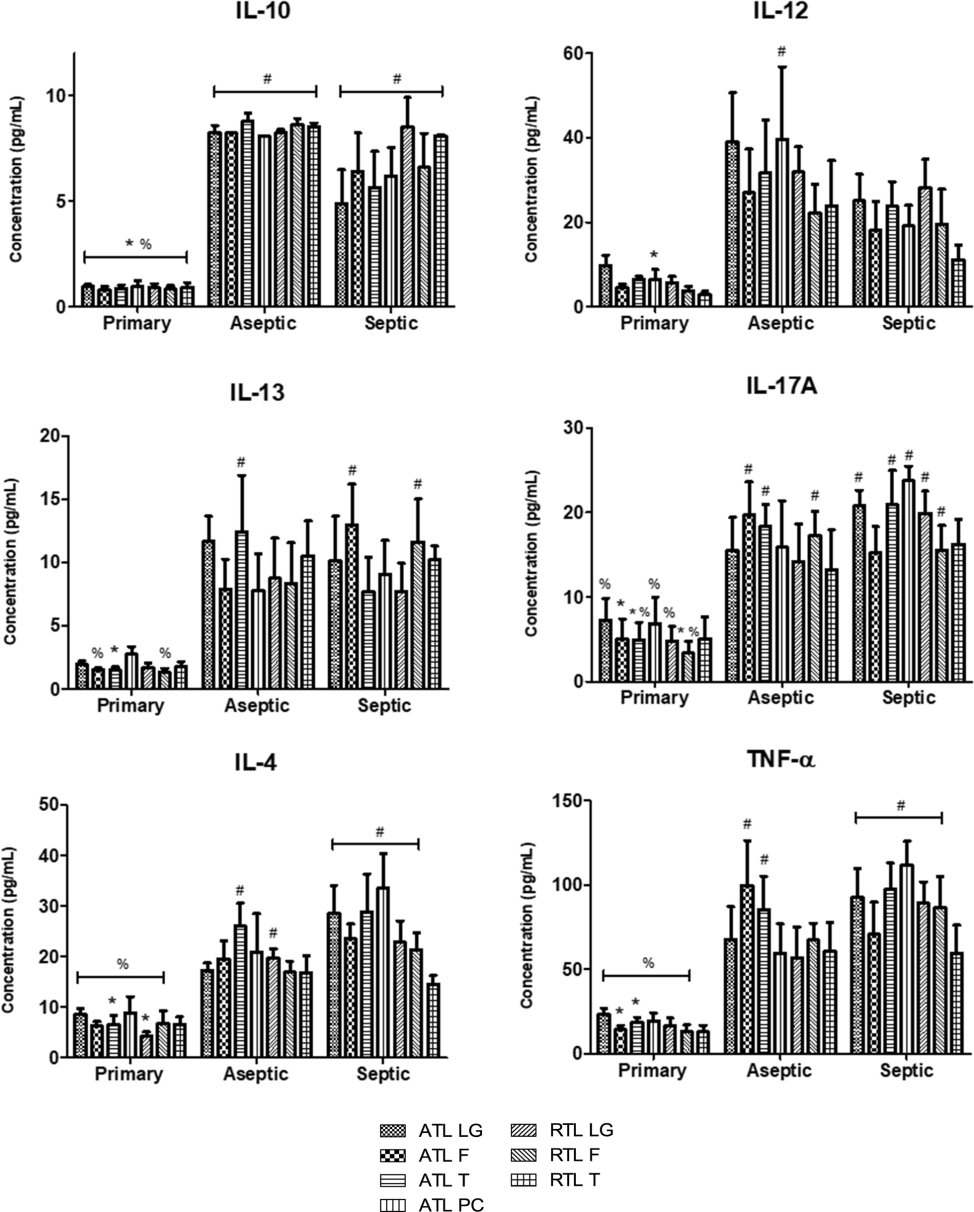
Six cytokines showed implant-related elevation in localized tissues that was not infection-dependent. Individual tissue locations are shown for all groups. Two-way ANOVAs with Bonferroni’s post-test were conducted to test for significant differences between groups at each individual location (p < 0.05). Significant differences between groups at a particular location are marked as: * denotes significant difference from primary TKA (N = 6); # denotes significant difference from aseptic TKR (N = 5); % denotes significant difference from septic TKR (N = 6); all symbols denote significance at the p < 0.05 level

Six cytokines, IL-10, IL-12p70, IL-13, IL-17A, IL-4, and TNF-α, exhibited higher levels in TKR tissues as compared to primary TKA tissues at a minimum of one location (p < 0.05). In other words, there were significant differences (p < 0.05) between primary TKA and aseptic/septic TKR, but there were no significant differences between aseptic TKR and septic TKR. For IL-10, the average value in primary TKA was 0.9 pg/mL, 8.4 pg/mL in aseptic TKR, and 6.6 pg/mL in septic TKR. All locations showed significantly different comparisons to aseptic TKR and septic TKR (Fig. 3, p < 0.05). With the same general trend, IL-12p70 had an average of 5.7 pg/mL in primary TKA, 30.7 pg/mL in aseptic TKR, and 20.7 pg/mL in septic TKR. However, IL-12p70 only showed one statistically significant comparison (p < 0.05) at the ATL PC location between primary TKA and aseptic TKR (Fig. 3). For IL-13, the average in primary TKA was 1.8 pg/mL, 9.6 pg/mL in aseptic TKR, and 9.9 pg/mL in septic TKR. Locations ATL F, ATL T, and RTL F exhibited significant comparisons between groups (Fig. 3, p < 0.05). Following this trend, IL-17A average concentrations were 5.3 pg/mL in primary TKA, 16.3 pg/mL in aseptic TKR, and 18.9 pg/mL in septic TKR. All locations except RTL T showed significant comparisons (Fig. 3, p < 0.05). For IL-4, average concentration in primary TKA was 6.9 pg/mL, which rose to 19.6 pg/mL in aseptic TKR, and further to 24.8 pg/mL in septic TKR. Again, all locations except RTL T showed significant comparisons (Fig. 3, p < 0.05). Finally, TNF-α followed the same trend, with an average concentration of 16.9 pg/mL in primary TKA, 71.1 pg/mL in aseptic TKR, and 86.8 pg/mL in septic TKR. All locations except RTL T showed significant comparisons (Fig. 3, p < 0.05).

### Quadratic discriminant analysis (QDA) revealed distinct cytokine profiles for TKA vs. TKR

**Fig. 4:**
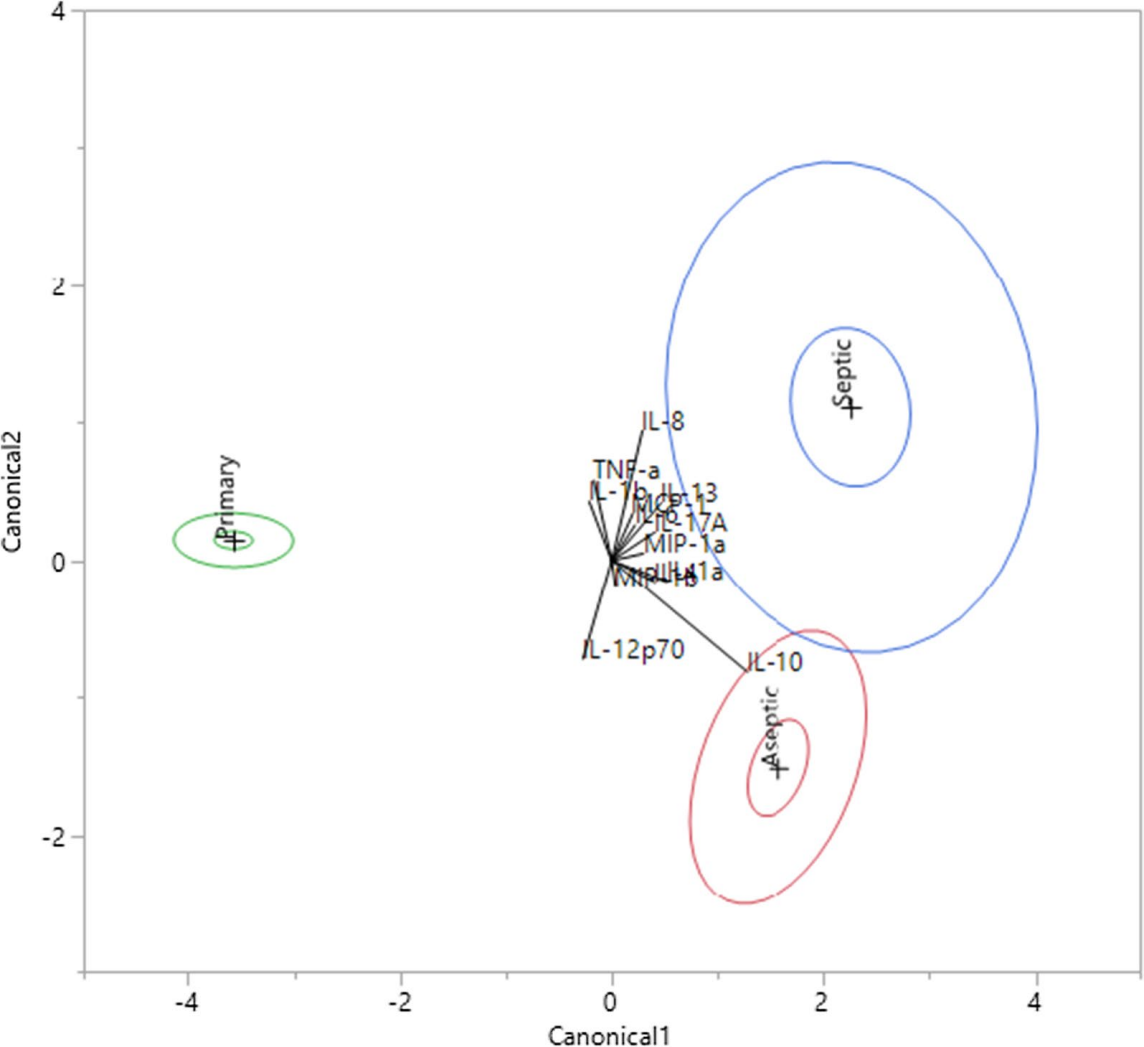
Quadratic discriminant analysis (QDA) revealed distinct groupings for primary TKA vs. TKR (aseptic or septic). Cytokines with significant infection-dependent or implant-related elevations via two-way ANOVA were analyzed via quadratic discriminant analysis. Canonical scores for each cytokine (covariate) were calculated, and the 95% confidence interval is shown for primary TKA (green), aseptic TKR (red), and septic TKR (blue). The + symbol represents the mean of each group. Biplot rays describe the degree of association of a certain cytokine with canonical variables

The two-way ANOVA comparisons of cytokines between different groups revealed seven cytokines that showed infection-specific elevation (beyond inflammation caused by implants), and six cytokines that showed increases due to implants, but not infection (Figs. 2 and 3). To further probe the structure of these cytokine profiles between groups, quadratic discriminant analysis was conducted. These 13 cytokines were included as covariates. The analysis classified the combined observed responses into pre-determined groups of primary TKA, aseptic TKR, and septic TKR. The group was predicted based on the covariate responses associated with each group, respectively. For each group, all seven locations were included for all individuals in that group, which means there were 42 counts for primary TKA (7 tissue locations, 6 patients), 35 values for aseptic TKR (7 tissue locations, 5 patients), and 42 counts for septic TKR (7 tissue locations, 6 patients). In total, of 119 counts, only 8 were misclassified, indicating a good prediction ability of the model. All 8 misclassifications were errors of a prediction of aseptic TKR group, when the values were originally from the septic TKR group. In other words, these individuals were falsely classified as aseptic based on cytokine profiles while they were actually septic. Further, there is overlap between the 95% confidence intervals for cytokine profiles of aseptic TKR and septic TKR patients (Fig. 4), which may be responsible for the misclassification.

### Effects of age, sex, and BMI on cytokine concentrations

The research presented here did not control for age, sex, or BMI due to the limited sample size of this exploratory study. To better understand the connections between cytokines of interest (IL-1α, IL-1β, IL-6, IL-8, MCP-1, MIP-1α, MIP-1β, IFN-γ, IL-10, IL-13, IL-17A, IL-4, and TNF-α) and these factors, Pearson correlations were run and analyzed for statistical significance. When the Bonferroni’s correction was applied, as described in [36], none of the correlations between cytokine levels and age, sex, or BMI were significant (p > 0.05), but the correlations are displayed in Table 2 for transparency. Although there is an established connection in the literature between inflammatory cytokine levels and age, sex, and BMI, the lack of significant Pearson correlation p-values indicates these were not confounding variables for this study [37–39].

**Table 2:** Pairwise pearson correlation values between cytokine concentrations and age, sex, and BMI

Cytokine	Primary TKA			Aseptic TKR			Septic TKR		
	Age	Sex	BMI	Age	Sex	BMI	Age	Sex	BMI
IL-1α	0.00	0.47	0.28	0.20	0.22	− 0.54	− 0.15	0.25	− 0.05
IL-1β	− 0.19	− 0.06	− 0.04	− 0.30	− 0.30	0.08	0.03	0.26	0.05
IL-6	0.00	− 0.09	− 0.12	0.03	− 0.19	− 0.21	0.00	0.22	− 0.02
IL-8	0.03	0.59	0.25	0.06	− 0.07	− 0.32	− 0.13	0.14	− 0.14
MCP-1	0.04	0.13	0.14	− 0.18	− 0.44	0.45	− 0.07	0.20	− 0.15
MIP-1α	− 0.10	0.13	− 0.04	− 0.05	− 0.30	0.09	0.33	0.08	− 0.13
MIP-1β	− 0.04	0.19	0.03	0.23	− 0.06	− 0.38	0.21	0.13	− 0.16
IL-10	− 0.26	0.29	0.17	− 0.31	0.00	− 0.20	− 0.11	− 0.25	0.30
IL-12p70	− 0.06	0.02	0.03	− 0.03	0.49	− 0.32	− 0.30	− 0.18	0.33
IL-13	− 0.19	0.49	0.28	− 0.06	0.41	− 0.06	− 0.22	− 0.23	0.22
IL-17A	0.00	− 0.08	− 0.31	0.31	0.85	− 0.45	− 0.34	0.06	0.32
IL-4	− 0.32	0.20	0.29	0.34	0.35	− 0.55	0.21	0.02	− 0.11
TNF-α	− 0.22	0.34	0.25	0.45	0.72	− 0.38	− 0.45	− 0.12	0.25

The pairwise correlation values are listed for each of the three groups: primary TKA, aseptic TKR, and septic TKR. Pearson correlation values are rounded to two decimal places. No correlations were found to be significant at the p < 0.05 level after Bonferroni’s correction

## Discussion

Inflammation in response to implants and infection following TKA/TKR procedures remains a serious complication and is a high priority for clinicians. However, not much is known about the local immune response of the tissue surrounding the implant/infection. While a variety of cytokines (and other biomarkers) have been researched from a systemic view [40, 41], their clinical use is still debated [31–33, 42]. Further, the cytokine responses have not been as well characterized on a localized tissue level. The tissue-level cytokine response may add further understanding of the localized environment and could give insight into tissue health that would aid clinicians in the management of these post-surgical complications through surgical debridement. Tissue-level cytokines have been measured with respect to spatial gradients in traumatic injury [16–18], respiratory infection [19], stroke [20], and allergic response [21], and these studies provided useful information regarding the respective immune responses. These have established a basis for this study to investigate the localized implant-related and infection-specific tissue responses.

This study focused on defining the tissue-level cytokine environment and modulation in response to implants and infection across several anatomical locations. Many human inflammatory cytokines have been implicated in the systemic response to implants (i.e., in serum/plasma) [43–45] and now aid in diagnosis of infection [46, 47]. However, this investigation is the first, to our knowledge, to assess multiple tissue locations surrounding the joint to address implant-related vs. infection-specific responses. Seven cytokines were identified as infection-specific, showing elevated concentrations in the septic TKR cohort compared to both the aseptic TKR and primary TKA cohorts: IL-1α, IL-1β, IL-6, IL-8, MCP-1, MIP-1α, and MIP-1β (p < 0.05). Several of these cytokines have illustrated their utility in the literature for diagnosis of PJI (i.e. IL-1α, IL-1β, IL-6, IL-8), but this is the first instance of their investigation on a tissue level [30–33]. Generally, these seven cytokines were elevated in ATL layer tissues compared to RTL layer tissues, which brings to light the importance of proximity to joint in dictating cytokine response. Pro-inflammatory cytokines like IL-1α, IL-1β, IL-6, and IL-8 have been noted for their roles in early infection response, producing a warning signal of pathogen invasion, and this response was present in septic TKR tissues [48, 49]. These early cytokine indicators recruit factors like MCP-1, MIP-1α, and MIP-1β that propagate the response to pathogens through Th1 and Th2 immune signaling cascades [50, 51]. Six cytokines were identified as exhibiting a response due to implantation, with elevations in aseptic and septic TKR vs. primary TKA: IL-10, IL-12p70, IL-13, IL-17A, IL-4, and TNF-α (p < 0.05). The elevated concentrations of these cytokines in localized tissues highlighted the degree of inflammation in implanted joints, without the presence of infection, which is likely due to the presence of a foreign body. The implant-related inflammation reflected less of the macrophage activation present in the septic TKR group, but exhibited elevation in anti-inflammatory cytokines like IL-10, IL-4, and IL-13 frequently associated with bone healing [7]. IL-17A and IL-12p70 have both pro- and anti-inflammatory roles, but the specific contributions to foreign body response are not well understood. Elevation of these cytokines, as well as TNF-α, implies there may be dysregulation of inflammatory response due to implant. These cytokines were not significantly elevated in infection at the p < 0.05 level, so they may be considered as indicators of aseptic or chronic inflammation that could be addressed with future research associated with TKA. Further, QDA analysis illustrated that cytokine profiles are distinct between all three cohorts, but there is significant overlap in the 95% confidence intervals of aseptic TKR and septic TKR. While there are several cytokines that distinctly separate these two cohorts, this analysis indicated that the degree of inflammation experienced between these groups is comparable. This finding agrees with the clinical decision to address inflammation and perform revision surgery, and these markers (IL-10, IL-12p70, IL-13, IL-17A, IL-4, and TNF-α) may show promise as helpful diagnostic monitoring markers for patients suffering from inflammatory complications in the absence of infection.

While this study had several limitations (i.e., single operating surgeon, heterogeneous cohort of patients, pathogen variability), it represents a novel characterization of tissue-level cytokines across different anatomical locations in response to implants as well as infection-specific inflammation. Further, these cytokines may give insight into the health of localized tissue following these procedures. Additionally, it highlights the utility of investigating a truly localized view of tissue health, by testing tissues surrounding the joint following these procedures; this approach could aid clinicians’ understanding of the localized tissue to better support clinical decision making. At the time of publication, all patients had reached at least the 1-year post-operative follow up without need for revision, with no recurrent infections, and the predictive value of these cytokines for successful surgical outcomes is of interest in future studies. These cytokines could potentially be incorporated to intra-operatively assess the amount of inflammation during surgery, providing information in real time about the viability of tissues for debridement. A more focused investigation of infection-specific markers IL-1α, IL-1β, IL-6, IL-8, MCP-1, MIP-1α, and MIP-1β could provide insight into the power of these cytokines to discriminate aseptic vs. septic tissues.

## Conclusions

In conclusion, this exploratory pilot study identified several cytokines that exhibited higher concentrations in response to implant-related and infection-specific post-operative inflammation. Some of these cytokines have been previously implicated in chronic inflammation and infection following TKA/TKR on a systemic level [11, 12, 30–33], and this study confirmed this trend on a localized tissue level. Literature has already illustrated that local inflammation is much more important for early post-operative recovery for a few markers [6], and this study expanded on that knowledge to provide an extended view of inflammatory cytokines involved in tissue health. Future studies will build off this localized tissue-level information to investigate the mechanisms of dysregulation observed between the groups.

Overall, investigating the localized tissue-level cytokines to understand implant-related and infection-specific inflammatory complications following knee arthroplasty may offer insight into localized response and provide new diagnostic and therapeutic options. Although this study did not control for age, sex, or BMI, these cytokines were not significantly correlated to these variables, suggesting these were not confounding factors (Table 2) in this study. Future work will focus on studies to include a larger cohort of patients to control for a variety of factors, including age, sex, BMI, and comorbidities. Ultimately, this study provided a basis to study these cytokines in surgical scenarios as a quantitative means to verify clinical decisions. More research is needed to confirm potential localized biomarkers that may be associated with chronic inflammation. In the future, larger cohort studies could utilize the infection-specific biomarkers for retrospective review of patient outcomes throughout the rehabilitation process.

## Data Availability

The datasets generated and/or analyzed during the current study are not publicly available to protect the privacy of the participants but are available from the corresponding author on reasonable request.

## Abbreviations

TKA: Total knee arthroplasty
TKR: Total knee revision
PJI: Prosthetic joint infection
IL: Interleukin
TNF-α: Tumor necrosis factor-alpha
IRB: Institutional Review Board
BMI: Body mass index
MSIS: Musculoskeletal Infection Society
MSSA: Methicillin-sensitive *Staphylococcus aureus*
CRP: C-reactive protein
ATL: Adjacent tissue layer
RTL: Radial tissue layer
F: Medial femoral condyle
T: Medial tibial plateau
LG: Lateral gutter
PC: Posterior capsule
PBS: Phosphate-buffered saline
4-PL: Four-parameter logistic
5-PL: Five-parameter logistic
LLOQ: Lower limit of quantitation
ULOQ: Upper limit of quantitation
IQR: Interquartile range
ANOVA: Analysis of variance
SEM: Standard error of the mean
MCP-1: Monocyte chemoattractant protein-1
MIP-1α: Macrophage inflammatory protein-1 alpha
MIP-1β: Macrophage inflammatory protein-1 beta
GM-CSF: Granulocyte–macrophage colony-stimulating factor
IFN-α: Interferon-alpha
IFN-γ: Interferon-gamma
IP-10: Interferon gamma-induced protein 10
QDA: Quadratic discriminant analysis
